# Ultraconserved Elements Sequencing as a Low-Cost Source of Complete Mitochondrial Genomes and Microsatellite Markers in Non-Model Amniotes

**DOI:** 10.1371/journal.pone.0138446

**Published:** 2015-09-17

**Authors:** Fábio Raposo do Amaral, Leandro G. Neves, Márcio F. R. Resende, Flávia Mobili, Cristina Y. Miyaki, Katia C. M. Pellegrino, Cibele Biondo

**Affiliations:** 1 Universidade Federal de São Paulo, Instituto de Ciências Ambientais, Químicas e Farmacêuticas, Laboratório de Genética Evolutiva, Rua Professor Artur Riedel, 275, Diadema, SP, 09972–270, Brazil; 2 RAPiD Genomics, LLC, 747 SW 2nd Avenue, Gainesville, FL, 32601, United States of America; 3 Universidade de São Paulo, Departamento de Genética e Biologia Evolutiva, Rua do Matão, 277, Cidade Universitária, São Paulo, SP, 05508–090, Brazil; 4 Universidade Federal do ABC, Centro de Ciências Naturais e Humanas, Rua Arcturus 03, Jardim Antares, São Bernardo do Campo, SP, 09606–070, Brazil; Temasek Life Sciences Laboratory, SINGAPORE

## Abstract

Sequence capture of ultraconserved elements (UCEs) associated with massively parallel sequencing has become a common source of nuclear data for studies of animal systematics and phylogeography. However, mitochondrial and microsatellite variation are still commonly used in various kinds of molecular studies, and probably will complement genomic data in years to come. Here we show that besides providing abundant genomic data, UCE sequencing is an excellent source of both sequences for microsatellite loci design and complete mitochondrial genomes with high sequencing depth. Identification of dozens of microsatellite loci and assembly of complete mitogenomes is exemplified here using three species of *Poospiza* warbling finches from southern and southeastern Brazil. This strategy opens exciting opportunities to simultaneously analyze genome-wide nuclear datasets and traditionally used mtDNA and microsatellite markers in non-model amniotes at no additional cost.

## Introduction

Mitochondrial DNA and microsatellites have been the markers of choice since the emergence of molecular data in studies of ecology and evolution [1–2]. Sequencing of mitochondrial DNA and isolation of microsatellites have relied for a long time on classic methods such as Sanger sequencing of amplicons [3], library enrichment [4] or cloning [5]. More recently, next-generation sequencing (NGS) has replaced Sanger sequencing in both prospection of microssatelite loci [6] and assembly of mitogenomes [7] due to its higher efficiency and lower costs per base pair.

The benefits of using multilocus data in estimates of historical demography, population structure and kinship have received strong theoretical and empirical support [8–12]. Recent emergence of massively parallel sequencing associated with techniques of genome reduction provide a cost-effective method to acquire large numbers of independent loci in a short timeframe. Consequently, studies based on techniques such as RAD-seq and sequence capture are becoming increasingly common [13–14]. Despite rapid increase in use of genomic data in evolutionary studies, mtDNA and microsatellite markers are still commonly used, and they will likely be employed in biodiversity studies along with NGS data for years to come.

Among the protocols currently used to obtain genomic variation, next-generation sequencing of ultraconserved elements has become a major source of data for animal phylogenetics in the last half decade. Nuclear ultraconserved elements (referred to henceforth as “UCEs”) are genomic regions first discovered in humans and which are highly conserved (>95% of identity) across distantly related organism, such as mammals, birds and fishes [15]. Such regions have been recently adopted as molecular markers for phylogenetic studies [16] due to their universality, which allows sequencing of thousands of homologous markers from sets of species with little genomic resources available. In addition, despite their conservatism, UCE flanking regions may contain sufficient information to study both deep [17] and shallow divergences [18]. The high similarity of UCE sequences allows the same set of target regions to be used across a number of distantly related amniotes [16] and more recently, fishes [19] and arthropods [20]. Despite its targeted nature, sequence capture is not 100% specific, and sequences located outside the target regions—called off-target sequences—may reach levels as high as 60% of all sequences obtained using commercial exome-capture kits [21,22]. Those unexpected sequences may be assembled, for example, into large mtDNA contigs [18], sometimes being sufficient to assemble an entire mitogenome (as briefly mentioned with UCEs in [23] and [24]). Mitogenomes are routinely assembled in human exome studies [25–27], but are still little explored subproducts of UCE studies.

Here we show in detail that the increasingly common sequence capture of UCEs may be used simultaneously as a source of dozens to hundreds of microsatellite loci and mitogenomes with high sequencing depth, rendering UCE sequencing a low-cost, hybrid strategy to obtain traditional markers alongside powerful genomic data. We use as exemplars three species of *Poospiza* warbling finches from the subtropical Brazilian Atlantic Forest, from which we obtained dozens of microsatellite loci and complete mtDNA genomes.

## Material and Methods

### Tissue sampling

We included three samples in this study, which represent one sample from each of three species of warbling-finches: the Gray-Throated Warbling Finch (*Poospiza cabanisi*), Buff-Throated Warbling Finch (*Poospiza lateralis*) and Bay-chested Warbling Finch (*Poospiza thoracica*). These species inhabit montane and subtropical forests in SE and S Brazil, and they are being studied as part of a larger phylogeography study (Amaral *et al*. in prep). A recent phylogenetic study [28] including two of these species (*P*. *thoracica* and *P*. *cabanisi*) suggests that they are not close relatives, and that *Poospiza* may be polyphyletic. *Poospiza lateralis* and *P*. *cabanisi*, in turn, appear to have split recently, and their divergence may be among the most recent in the Neotropics (Amaral *et al*., unpublished data). *Poospiza lateralis* and *P*. *cabanisi* are allopatric, while *P*. *thoracica* is sympatric to both. Specimens were attracted in the field using playback recordings and collected using an air shotgun [29]. This research has been approved by the ethics committee of the Federal University of São Paulo (CEP 0069/12), including the adopted sampling procedure. Field expeditions and specimen collection permits for all visited localities were granted by ICMBio (permit numbers 14673 and 30840). All specimens were housed at the ornithological collection of the Museu de Zoologia da Universidade de São Paulo (MZUSP), and tissue samples deposited at the Laboratório de Genética e Evolução de Aves da Universidade de São Paulo (LGEMA-USP, see Table 1).

**Table 1:** Sample information and descriptive sequence statistics.

	*Poospiza cabanisi*	*Poospiza lateralis*	*Poospiza thoracica*
Field number	FRA67	FRA46	SC04
Locality	Pelotas, RS, Brazil	Dores do Rio Preto, ES Brazil	Urubici, SC, Brazil
Coordinates	-31.5265, -52.5505	-20.5125; -41.8075	-28.1449; -49.6324
Read pairs	2527058	3105898	2935389
Contigs assembled	8638	11392	9365
UCE loci captured	625	632	626
Mitogenome length (bp)	16768	16773	16772
Mitogenome GC content (%)	47.1	47.1	46.6
mtDNA Sequencing depth (X) with duplicates (Mean, st. dev., min, max)	1161, 263, 37, 2258	1081, 294, 9, 2419	588, 138, 32, 1083
mtDNA Sequencing depth (X) without duplicates (Mean, st. dev., min, max)	95, 10, 6, 122	74, 9, 4, 95	81, 11, 2, 112
Number of microsatellite loci (total / with primer designed)			
Dinucleotide	250 / 54	307 / 57	277 / 52
Trinucleotide	85 / 6	105 / 15	87 / 13
Tetranucleotide	23 / 4	30 / 3	27 / 3
Pentanucleotide	2 / 0	6 / 0	4 / 0
Hexanucleotide	1 / 0	3 / 2	2 / 0
All repetition types	361 / 64	451 / 77	397 / 68

MZUSP and LGEMA numbers pending. “Read pairs” refer to number of pairs resulting from resynchronization of files processed with Illumiprocessor.

### Laboratory methods

We extracted total DNA from muscle samples using the Qiagen DNeasy kit (Valencia, CA) according to the manufacturer's protocol, including the suggested RNAse treatment. Sequence capture and sequencing of ultraconserved elements were performed according to the original UCE capture and sequencing protocols [16] by RAPiD Genomics (Gainesville, FL, USA). Modifications made to the original protocol consisted of the reduction from 2,560 to 650 probes (targeting 634 loci selected to cover all chromosomes of the *Gallus* genome), use of 100 bp paired-end Illumina Hiseq 2000 sequencing run, and use of 16 cycles in both pre- and post-capture PCR reactions. We sequenced the three specimens used here in a multiplexed batch of 48 individuals (i.e. sequencing included 45 individuals from other studies).

### Sequence quality control and assembly

We separated raw sequences by individual tags using Illumina's Casava software. Sequences were initially evaluated using FastQC 0.10.1 [30]. Adapters, barcodes and low quality regions were removed using Illumiprocessor 2.0.7 [31], which allows processing of Illumina sequencing reads using the trimming tool Trimmomatic 0.32.1 [32]. The resulting reads were processed using Phyluce 1.4 [33], which is a software package that allows processing of trimmed reads into aligned loci. We performed processing in Phyluce up to the assembly stage, using the Trinity assembler v20140717 [34]. All analyses were performed using default parameters. Contigs resulting from assembly were used for downstream analyses.

### mtDNA assembly, variant calling, annotation and phylogenetic inference

Contigs were compared to all avian mtDNA genomes at NCBI as of August 6^th^ 2014 using the standalone version of BLAST 2.2.28 [35]. Contigs with a strong match (e-value < 0.1) to avian mtDNA were used to manually assemble the mitogenome in Bioedit 7.1.3 [36]. Despite the haploid nature of mtDNA, we called SNPs in order to detect potential heteroplasmy and/or PCR errors. Paired reads processed in Illumiprocessor were resynchronized using custom scripts and then mapped to the respective mitogenome using Bowtie2 v2.2.4 [37]. PCR duplicates, which consist of identical sequences that arise due to amplification during library preparation, were removed using Samtools 0.1.19 [38], and variants and indels were called with Freebayes 0.9.15 [39] using the diploid option and standard filters (-m 30-q 20-R 0-S 0). Sequencing depth statistics were obtained using Bedtools 2.17 [40].

Complete mitochondrial genomes were annotated based on the automatic methods of MITOS [41] and DOGMA [42] using default parameters. Incongruences in the results obtained between the two methods for each species were manually adjusted based on comparisons to the annotated mtDNA genomes of *Gallus gallus* (NC_001323) and *Emberiza pusilla* (NC_021408). Final annotation of the mitogenomes was performed using Geneious 6.1.8 [43].

In order to evaluate mitochondrial sequence authenticity, we performed a phylogenetic analysis including cytochrome b sequences from the mitogenomes obtained here and public sequences from species attributed to the Poospizinae sub-family [28], which were sequenced by those authors using standard Sanger sequencing. We included representatives of the genera *Poospiza*, *Compsospiza*, *Cnemoscopus*, *Cypsnagra*, *Donacospiza*, *Hemispingus*, *Nephelornis*, *Piezorina*, *Pyrrhocoma*, *Thlypopsis*, *Urothraupis and Xenospingus*. We generated sequence alignments in Muscle [44] and performed model selection based on the Akaike Information criterion as implemented in MrModeltest 2 [45], which indicated HKY+I+G as the best-fit model. We performed Bayesian phylogenetic inference in MrBayes 3.2.3 [46] using the CIPRES infrastructure [47], adopting a conservative burnin of 25% and *Xenospingus concolor* as outgroup. MrBayes runs were performed twice to verify convergence of topology and posterior probabilities obtained from independent runs.

### Identification of microsatellite loci candidates

Contigs resulting from the Trinity assembler (implemented in Phyluce) were used as input for QDD 1.3 [48], which is a software package that detects microsatellites and allows primer design. We searched for pure di-, tri-, tetra-, penta- and hexa-nucleotides with at least five repeats. Potential primers were designed using default settings in Primer3 [49] as implemented in QDD. Sequences used for primer design were compared to the corresponding species’ mitogenome using BLAST, and removed in case of matches. The same procedure was also performed using sex chromosomes in order to identify sex-linked microsatellites. Comparisons to sex chromosomes were based on both chicken and zebra finch Z chromosomes, while only the chicken W chromosome was used due to the lack of currently available zebra finch W chromosome sequences. Identification of homologous loci across species was based on alignment of sequences used for primer design in Geneious. Alignments with a minimum of two species were used to evaluate microsatellite polymorphism.

## Results and Discussion

### Mitogenomes

Here we show that UCE sequencing may be a low-cost (~ US$ 100 per sample) and efficient method for obtaining high-depth complete mitogenomes based solely on off-target sequences, with no need for mtDNA-specific baits. The three samples sequenced here provided complete mitogenomes along with at least 625 out of 634 target loci per sample (Table 1). Mitogenome sequencing depth was high in all cases (Table 1), with means higher than 500x when PCR duplicates were considered. Removal of the PCR duplicates considerably dropped depth, but mean depths remained high (>70x), with depth ranging from 2x to 122x. Use of skeletal muscle as source material, which has a high mtDNA/nDNA ratio, may be responsible for such high mean depths [25,50], and further tests will be important to evaluate the efficiency of this protocol with other types of tissue. Blood samples, for example, appear to be a poor source of mtDNA off-target in UCE experiments (B. Faircloth, personal communication). Our results in particular showed PCR duplication levels higher than 90% for all three species, and those values may be considerably lowered by decreasing the number of PCR cycles during library amplification (B. Faircloth, personal communication).

Mitogenome completeness of the three warbling finches is supported by the presence of all regions found in two close mitogenomes available (*Thraupis episcopus*, [51]; *Emberiza pusilla*, [52]), including one control region, two rRNAs, 22 tRNAs and 13 protein-coding genes (S1 Table). Gene synteny was observed among the three species (S1 Table), and their gene order is the one present in most oscines studied so far. Genome sizes and G+C contents were also similar to those of published avian mitogenomes, ranging between 16,768–16,773 bp and 46.6–47.1%, respectively. Five mitochondrial polymorphic sites were found (*P*. *lateralis*, T and C at position 7847 and A and T at position 15290; *P*. *cabanisi*, C and A at position 15600; *P*. *thoracica*, A and T at position 14, and T and C at position 15606), and they can be explained by PCR error or heteroplasmy, which is an increasingly common finding in mtDNA studies [53–55]. Although an early diverging numt could also explain these polymorphic sites [56], we expect that use of muscle as DNA source makes this hypothesis unlikely due to its high mtDNA/nDNA ratio. Bayesian phylogenetic inference based on cytochrome b sequences also supported authenticity of the mitochondrial sequences with maximum posterior probabilities (S1 Fig). *Poospiza lateralis* was sister to *P*. *cabanisi*, a finding in line with their conspecificity according to some authors (see [57] for a taxonomic review). In addition, both sequences of *P*. *cabanisi* and *P*. *thoracica* collected here were sister to conspecific sequences obtained from Genbank.

### Microsatellite loci

We found a minimum of 361 microsatellites per species (Table 1), with primer design being possible for at least 64 loci per species (S1 Table). These loci numbers are higher than those recommended by Gardner *et al*. [58] for typical molecular ecology studies (~40 designable, unique loci). We believe that the number of microsatellites for which primers cannot be designed may decrease with use of inserts larger than the ones used here (~300 bp), which could translate into higher average assembly length and consequently longer flanking regions for primer design. We found microsatellite loci in both UCE and non-UCE data. Purifying selection may be a concern in UCE regions [59], which may include linked microsatellite loci. However, less than one fifth of the loci with flanking sequence suitable for primer design found here matched UCEs (*P*. *cabanisi*: 11 of 64; *P*. *lateralis*: 11 of 77; *P*. *thoracica*: 10 of 68). No mitochondrial or W-linked microsatellite loci were found, but a minor proportion (eight in *P*. *cabanisi*, eight in *P*. *lateralis*, and nine in *P*. *thoracica*) were Z-linked. From the microsatellite loci alignable between at least two species (23), 10 matched UCE loci and 13 consisted of non-UCE loci, with one of each category being variable. Although the small panel of individuals (2–3) may not be sufficient to reliably explore polymorphism, most of the sequences found were outside UCE regions, and thus should present levels of variation similar to those of microsatellite loci isolated using standard techniques (e.g. microsatellite enrichment).

### Advantages and potential pitfalls

Obtaining mitogenomes and microsatellite loci from off-target sequences is an opportunistic approach that depends on the incomplete efficiency of sequence capture protocols. Thus, future increases in sequence capture efficiency can negatively affect any application based on off-target sequences. However, despite the rising efficiency in sequence capture, off-target sequences are still common (e.g. protocols with only 40% on target in some cases, [26]), providing abundant mtDNA sequences and microsatellite loci. Even in cases of high specificity, increasing sequencing depth may still provide sufficient off-target sequences for diverse applications at reasonable costs. When the intended number of microsatellite loci or the desired mitogenome completedeness is not met, the low cost associated with UCE sequencing (~ US$ 100/sample) makes replicated sequencing of the same sample or sequencing of multiple individuals of a given species still a cost-effective strategy. Use of mixed mtDNA and nuclear baits (as suggested by Falk et al. [60]) is certainly an exciting and promising alternative to simultaneous sequencing of mtDNA and UCEs, but may also have its downsides, such as the additional costs of design and synthesis of compatible baits at different concentrations. In addition, designing baits for fast-evolving mitogenomes may be challenging when sequencing distant species, which is a common requirement of phylogenetic studies. Finally, use of off-target sequences may facilitate locus design of non-model organisms, since they contain non-UCE microsatellite markers.

## Conclusion

Here we show that the increasingly common sequencing of UCE loci may be a low-cost and simultaneous source of mitogenomes and sequences for microsatellite primer design with good sequencing depth. Since UCE sequencing has been successfully used in many non-model species of amniotes—and more recently, fishes and arthropods—the mitogenomes and microsatellite loci can be obtained for thousands of animal species using the strategy presented here, including species with limited genomic resources available.

## Supporting Information

S1 Table Characterization of the mitogenomes of *Poospiza cabanisi*, *P*. *lateralis* and *P*. *thoracica*. (XLS)

S2 Table List of microsatellite with designable primers. (XLS)

S1 Fig Bayesian phylogeny of Poospizinae thraupids based on cytochrome b sequences. Bold names with asterisks indicate sequences obtained here. Numbers on branches correspond to posterior probabilities.
(TIF)
